# A novel conditioning regimen of chidamide, cladribine, gemcitabine, and busulfan in the autologous stem cell transplantation of aggressive T-cell lymphoma

**DOI:** 10.3389/fonc.2023.1143556

**Published:** 2023-03-09

**Authors:** Qiang Zeng, Hang Zhang, Pu Kuang, Jian Li, Xinchuan Chen, Tian Dong, Qiuhui Wu, Chuanli Zhang, Chunping Chen, Ting Niu, Ting Liu, Zhigang Liu, Jie Ji

**Affiliations:** ^1^ Department of Hematology and Institute of Hematology, West China Hospital, Sichuan University, Chengdu, China; ^2^ Stem Cell Transplantation and Cellular Therapy Division, Clinic Trial Center, West China Hospital, Sichuan University, Chengdu, China; ^3^ Department of Hematology, West China Hospital/Shangjin Nanfu Hospital, Chengdu, China

**Keywords:** chidamide, cladribine, gemcitabine, busulfan, ASCT - autologous stem cell transplantation, T-cell lymphoma

## Abstract

**Background:**

The prognosis of patients with peripheral T-cell (PTCL) or lymphoblastic T-cell lymphoma (T-LBL) remains poor under current conditioning regimens before receiving autologous stem cell transplantation (ASCT).

**Methods:**

Patients with PTCL or T-LBL were enrolled to receive ASCT using the conditioning regimen of chidamide, cladribine, gemcitabine, and busulfan (ChiCGB). Positron emission tomography-computed tomography (PET/CT) was used to evaluate the response to ASCT. Overall survival (OS) and progression-free survival (PFS) were employed to assess the patient outcome, and adverse events were used to assess the regimen’s safety. The survival curve was estimated *via* the Kaplan-Meier method.

**Results:**

Twenty-five PTCL and 11 T-LBL patients were recruited. The median time to neutrophile and platelet engraftments was 10 days (8–13 days) and 13 days (9–31 days), respectively. The 3-year PFS and OS were 81.3 ± 7.2% and 88.5 ± 5.4% for all patients; 92.0 ± 5.4% and 81.2 ± 8.8% for PTCL patients; and both 81.8 ± 11.6% for T-LBL patients, respectively. The 3-year PFS and OS were both 92.9 ± 4.9% for patients with complete response (CR) but 50.0 ± 17.7% and 75.0 ± 15.3% for patients with non-CR, respectively. Infection was the most common non-hematological toxicity, and all toxicities were mild and controllable.

**Conclusions:**

ChiCGB was a potentially effective and well-tolerated conditioning regimen to improve the prognosis of patients with aggressive T-cell lymphoma. Future randomized controlled trials are needed to assess ChiCGB as a conditioning regimen for ASCT.

## Introduction

Peripheral T-cell lymphoma (PTCL) and lymphoblastic T-cell lymphoma (T-LBL) are rare and aggressive subtypes of non-Hodgkin lymphoma that exhibit chemotherapy insensitivity and poor prognosis. Clinical data on the two subtypes have shown 5-year progression-free survival (PFS) of 13–29% and 5-year overall survival (OS) of 15–64% after conventional chemotherapy (1, 2). Autologous stem cell transplantation (ASCT) is an essential treatment for aggressive T-cell lymphoma. It is frequently used as standard-of-care for patients in first complete response (CR1), despite the advent of new drugs. Several retrospective and prospective studies have demonstrated that ASCT significantly prolongs the survival time of patients with aggressive T-cell lymphoma (3, 4). However, the prognosis for patients with aggressive T-cell lymphoma remains unfavorable compared to other lymphomas.

The conditioning regimen used in ASCT is critical to the effectiveness of treatment, and different conditioning regimens may result in different outcomes even for the same disease. For instance, patients with Hodgkin lymphoma receiving carmustine, etoposide, cytarabine, and melphalan (BEAM) as a conditioning regimen had a 5-year PFS of 66% and a 5-year OS of 79%. In contrast, those receiving busulfan, cyclophosphamide, and etoposide (BUCYVP16) had a 5-year PFS of 33% and a 5-year OS of 54% (5). Standard conditioning regimens for T-cell lymphomas include BEAM, total body irradiation (TBI) with cyclophosphamide, cyclophosphamide, carmustine, etoposide (CBV), BUCYVP16, and others. These regimens have resulted in PFS rates of 33%–53%, which is markedly lower than the survival rates of patients with B-cell lymphomas undergoing ASCT (6–8). Therefore, selecting a conditioning regimen according to the characteristics of tumor cells may improve the efficacy of ASCT and the prognosis of patients.

In our previous work, we demonstrated that cladribine, gemcitabine, and busulfan (CGB) had a synergistic effect on killing lymphoma cells, and a histone deacetylase inhibitor (HDACi), vorinostat, sensitized the lymphoma cells to the CGB regimen by causing changes in the chromatin structure (9). Another HDACi, chidamide, inhibits HDAC 1, 2, 3, and 10 and is an oral agent approved by the National Medical Products Administration (NMPA) of China for refractory/relapsed PTCL, also synergized with CGB in killing lymphoma cells. Based on this work, we previously conducted a Phase 2 clinical trial on the ChiCGB conditioning regimen in ASCT for non-Hodgkin’s lymphoma, which showed that the regimen was effective and safe (10). Herein, building on our previous work, we further subdivided the disease categories and designed a single-arm, Phase 2 clinical trial to investigate whether the conditioning regimen of chidamide with CGB (ChiCGB) could improve the clinical efficacy compared to historical other conditioning regimens for aggressive T-cell lymphomas.

## Methods

### Study design

Our study was a single-arm, prospective, Phase 2 clinical trial registered on the Clinical Trial Registry (clinicaltrials.gov, NCT03602131). The primary endpoint was PFS. Other endpoints included OS, overall response rate, CR rate, relapse rate, and non-hematological adverse events graded by the National Cancer Institute Common Toxicity Criteria (NCI-CTC, Version 4.0).

All patients were reinfused with peripheral hematopoietic stem cells. PFS was defined as the period from ASCT to disease progression, relapse, death from any cause, or the last follow-up; OS was defined as the period from ASCT to death from any cause or the last follow-up. Positron emission tomography-computed tomography (PET/CT) was used to evaluate disease status prior to ASCT and the response to ASCT every three months in the first year and every six months after one year. According to the Lugano classification in 2013 (11), those with a Deauville score of 3 or less were determined to be effective, while those with a Deauville score of 4 or more were considered ineffective or relapsed.

### Patient population

After finishing planned chemotherapy, patients who met the following inclusion criteria were recruited for our study: (a) age ranged from 18 to 70 years old; (b) aggressive T-cell lymphomas including i) PTCLs other than ALK+ anaplastic large cell lymphoma (ALCL) in CR1, ii) T-LBL without BM involvement in CR1, iii) any chemo-sensitive relapsed PTCL in CR or partial response (PR); (c) adequate renal function, as defined by estimated serum creatinine clearance ≥ 50 ml/min and/or serum creatinine ≤ 1.8 mg/dL; (d) adequate hepatic function, as defined by serum glutamate oxaloacetate transaminase (SGOT) and/or serum glutamate pyruvate transaminase (SGPT) ≤ 3 times the upper limit of normal; serum bilirubin and alkaline phosphatase ≤ 2 times the upper limit of normal; (e) adequate pulmonary function with forced expiratory volume at one second (FEV1), forced vital capacity (FVC) and diffusing lung capacity for carbon monoxide (DLCO) ≥ 50% of expected after correction for hemoglobin; (f) adequate cardiac function with left ventricular ejection fraction ≥ 50% and no uncontrolled arrhythmias or symptomatic cardiac disease; and (g) negative Beta human chorionic gonadotropin (HCG) test in a woman with child-bearing potential, defined as not post-menopausal for 12 months or no previous surgical sterilization.

Of the patients who met the above criteria, those with the following conditions were excluded from the study: (a) central nervous system was involved; (b) relapse after stem cell transplantation; (c) active infection requiring parenteral antibiotics; (d) active hepatitis B or C(HBV DNA ≥ 10,000 copies/mL); (e) human immunodeficiency virus (HIV) infection, unless the current recipient of effective antiretroviral therapy with undetectable viral load and a normal cluster of differentiation 4 (CD4) counts; (f) evidence of either cirrhosis or Stage 3–4 liver fibrosis alongside chronic hepatitis C or positive hepatitis C serology; and (g) a corrected QT interval (QTc) longer than 500 ms.

### Treatment plan

The conditioning regimen of ChiCGB started with a 30mg dose of chidamide taken orally on Day -7. Subsequently, chidamide was given at the same dose one hour before chemotherapy on Days -4, -1, and +3. Then, a 6 mg/m^2^ dose of cladribine was given from Day -6 to Day -2, while a 2.5g/m^2^ dose of gemcitabine was given on Day -6 and Day -2, with a 4-hour infusion interval between the two drugs. Meanwhile, a 3.2mg/kg dose of busulfan was administrated intravenously for more than 3 hours after the finish of gemcitabine once daily from Day -6 to Day -3. Finally, hematopoietic stem cells were reinfused on Day 0 (**Table 1**). All patients received the netupitant/palonosetron capsules to prevent chemotherapy-induced nausea and vomiting (CINV).

**Table 1:** Conditioning regimen of ChiCGB.

Drugs	Dosage	-7	-6	-5	-4	-3	-2	-1	0	+3
**Busulfan**	3.2mg/kg		+	+	+	+				
**Cladribine**	6mg/m^2^		+	+	+	+	+			
**Gemcitabine**	2.5g/m^2^		+				+			
**Chidamide**	30mg	+			+			+		+
**Stem cells**									+	

ChiCGB, chidamide, cladribine, gemcitabine, busulfan.

### Statistical analysis

The sample size was estimated *via* the Weibull model. A previous study reported a PFS of 40% in aggressive T-cell lymphomas, while we estimated a PFS increase to 65% in our study (12). The sample size was calculated with 80% power and an overall 5% significance level using PASS 21.0 software (NCSS Statistical Software, Kaysville, Utah, USA). Considering the 10% drop-out rate during the follow-up period, the final calculated sample size required was 26 patients.

OS and PFS were employed to evaluate the prognosis of patients with T-cell lymphoma undergoing ASCT. The survival curve was estimated using the Kaplan-Meier method and compared *via* log-rank tests. Two-sided *P* values < 0.05 were considered significant. All statistical analyses were performed using GraphPad Prism 8.0 software (Graphpad Software, San Diego, CA, USA).

## Result

### Patient characteristics

**Figure 1:**
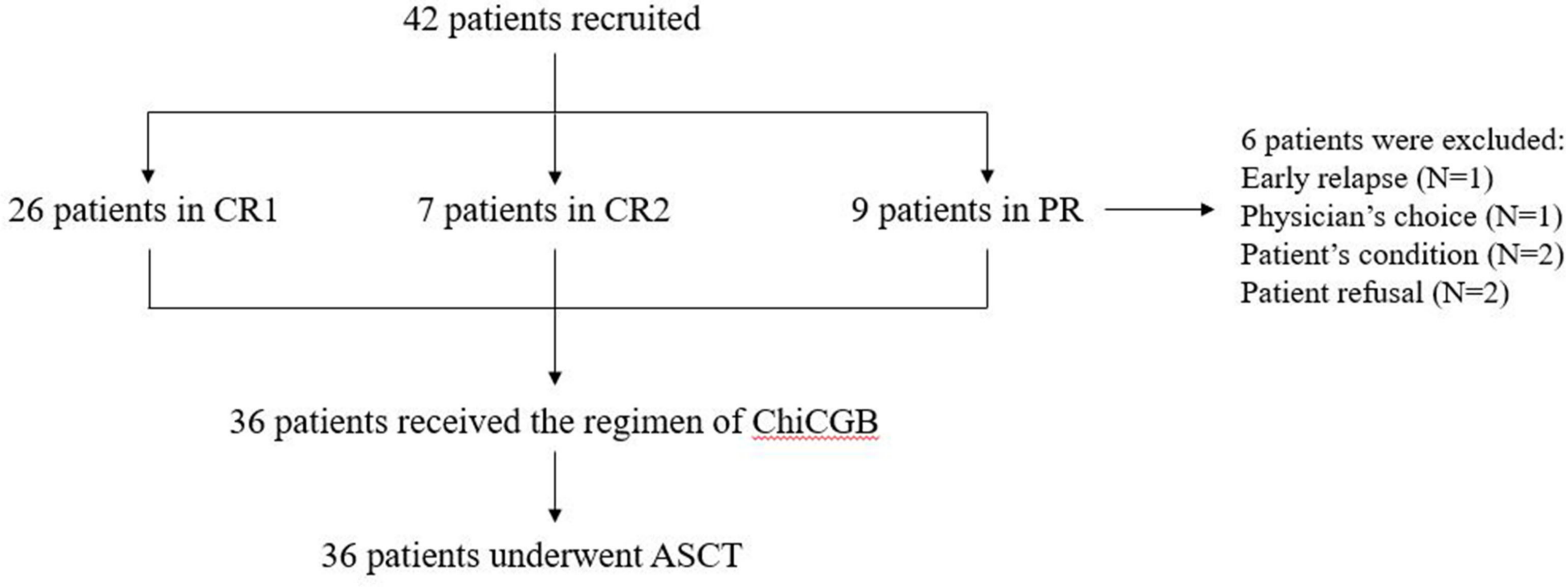
The flowchart of patient inclusion.

From April 2015 to April 2022, 42 patients with PTCL or T-LBL were recruited for our study after finishing the planned chemotherapy at West China Hospital. After inclusion in the clinical trial, 6 patients were excluded due to the patient’s refusal, early relapse, physician’s choice, and patient’s condition, and altogether 36 patients, with 10.2 months of median duration between diagnosis and ASCT, were eventually enrolled in our trials (**Figure 1**). Of these patients, 21 (58.3%) were male, and the median age was 47 years old (range: 18–66 years). Twenty-five patients (69.4%) were diagnosed with PTCL, and 11 (30.6%) with T-LBL. Among these patients, 22 (61.1%), including all T-LBL, achieved CR1, 6 (16.7%) achieved CR2, and 8 (22.2%) achieved PR prior to ASCT.

All hematopoietic stem cells for ASCT were mobilized and collected from peripheral blood within three months prior to ASCT. The median number of CD34^+^ cells in the graft was 2.98×10^6^/kg (range: 1.24–13.20×10^6^/kg). All patients were fully engrafted during hospitalization. The median time to neutrophile engraftment was 10 days (range: 8–13 days) while the median time to platelet engraftment was 13 days (range: 9–31 days). The remaining information is listed in **Table 2**.

**Table 2:** Characteristics of included patients.

Characteristics	Value (%)
Patients	36
Male	21 (58.3%)
Female	15 (41.7%)
Median age (Range, year)	47 (18-66)
Disease subtypes	
PTCL	
PTCL, NOS ALCL, ALK- ALCL, ALK+ AITL T-LBL	7 (19.4%) 5 (13.9) 4 (11.1) 9 (25.0%) 11 (30.6%)
Disease status prior to ASCT	
CR1	22 (61.1%)
CR2	6 (16.7%)
PR	8 (22.2%)
Ann arbor stage	
PTCL	
I	0
II	0
III	13 (36.1%)
IV	12 (33.3%)
T-LBL	
I II III IV	0 3 (8.3%) 3 (8.3%) 5 (13.8%)
CD34^+^ cells (Range, ×10^6^/kg)	2.98 (1.24-13.20)
Bone marrow infiltration	
Present	12 (33.3%)
Absent	24 (66.7%)
Extranodal infiltration	
Present	22 (61.1%)
Absent	14 (38.9%)
B symptoms	
Present	15 (41.6%)
Absent	21 (58.3%)
LDH > normal	8 (22.2%)
IPI score	
0	4 (11.1%)
1	14 (38.9%)
2	12 (33.3%)
3	6 (16.7%)
4	0
5	0
Median time to neutrophile engraftment (Range, day)	10 (8-13)
Median time to platelet engraftment (Range, day)	13 (9-31)
Median time of hospitalization (Range, day)	31 (21-55)

ASCT, autologous stem cell transplantation; PTCL, peripheral T-cell lymphoma; NOS, not otherwise specified; ALCL, anaplastic large-cell lymphoma; ALK, anaplastic lymphoma kinase; AITL, angioimmunoblastic T-cell lymphoma; T-LBL, T cell lymphoblastic lymphoma; CR, complete response; PR, partial response; LDH, lactate dehydrogenase; IPI, international prognostic index.

### Patient outcomes

**Figure 2:**
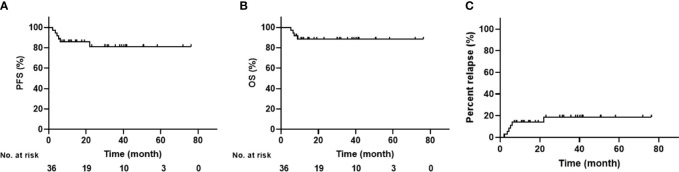
Survival Curve of All Included Patients **(A)** PFS; **(B)** OS; **(C)** Percentage of relapse).

The last follow-up time ended on November 1, 2022, and the median follow-up period was 30 months (range: 7.1–76.3 months). Overall, the 3-year PFS was 81.3 ± 7.2% and the 3-year OS was 88.5 ± 5.4%; neither medians was reached (**Figure 2**). All but one patient responded well to the treatment and were in CR in the third month after ASCT. The outlier patient died 6.9 months after relapse. Three patients relapsed at 3.5, 5.3, and 6.1 months, and all died within two months (**Figure 2**). All four patients died from disease progression. Another two patients relapsed but have survived to date. However, one patient with angioimmunoblastic T-cell lymphoma (AITL) experienced recurrent lymphadenopathy in the neck at 28 months post-transplantation. The pathological finding showed that the heterogeneous lymphocytes were of B-cell origin and EBV-positive, and EBV-DNA was detected in a blood sample, indicating the diagnosis of EBV-positive diffuse large B cell lymphoma (DLBCL). No sign of a T-cell lymphoma relapse was found in this patient. To date, the remaining patients have survived without relapse.

**Figure 3:**
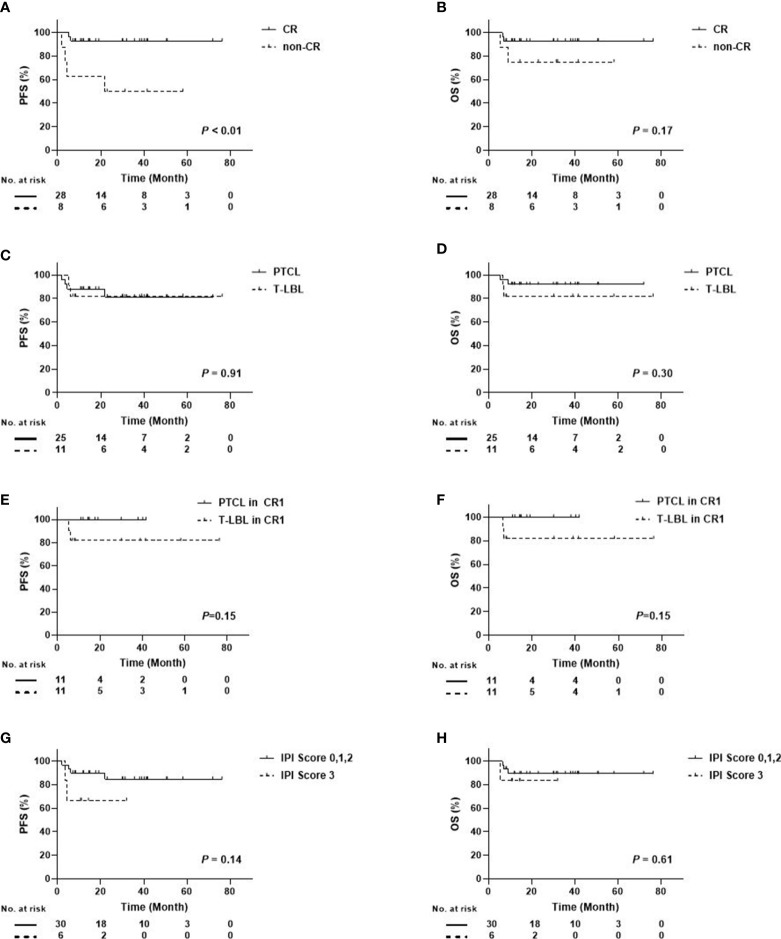
Survival Curves Between Different Subgroups in Patients with PTCL/T-LBL **(A)** PFS Between CR and Non-CR; **(B)** OS Between CR and Non-CR; **(C)** PFS Between PTCL and T-LBL; **(D)** OS Between PTCL and T-LBL; **(E)** PFS Between PTCL in CR1 and T-LBL in CR1; **(F)** OS Between PTCL in CR1 and T-LBL in CR1; **(G)** PFS Between IPI score 0, 1, 2, and 3; **(H)** OS Between IPI score 0, 1, 2, and 3).

In the subgroup analysis, disease status prior to ASCT, disease subtype, and IPI score were included. **Figure 3** shows that the 3-year OS and PFS for the patients with CR were 92.9 ± 4.9%, while those with non-CR were 75.0 ± 15.3% and 50.0 ± 17.7%. A difference occurred between CR and non-CR prior to ASCT regarding the PFS and OS. Especially in PFS, the difference was statistically significant (*P* < 0.01). No differences were found between PTCL and T-LBL (3-year OS: 92.0 ± 5.4% *vs*. 81.8 ± 11.6%, *P* = 0.30; 3-year PFS: 81.2 ± 8.8% *vs*. 81.8 ± 11.6%, *P* = 0.91), between PTCL in CR1 and T-LBL in CR1 (3-year OS: 100.0% *vs*. 81.8 ± 11.6%, *P*=0.15; 3-year PFS: 100.0% *vs*. 81.8 ± 11.6%, *P* = 0.15), and between IPI score 0, 1, 2, and 3 (3-year OS: 89.4 ± 5.8% *vs*. 83.3 ± 15.2%, *P*=0.61; 3-year PFS: 84.7 ± 7.3% *vs*. 66.7 ± 19.2%, *P* = 0.14) (**Figure 3**).

### Non-hematological toxicity associated with ChiCGB

The most common non-hematological adverse event caused by the new conditioning regimen of ChiCGB was infection (58.3%), including in the respiratory and gastrointestinal tract. The main manifestation of infection was fever and the incidence of neutropenic fever was 36.1%. Infection symptoms could be significantly improved after empirical anti-infection treatment. Furthermore, Epstein-Barr virus (EBV) reactivation and post-transplant lymphoproliferative disorders (PTLD) were not observed in all patients. Based on the NCI-CTC (Version 4.0), other toxicities of Grades 2 to 4 are displayed in **Table 3**. No Grade 4 or 5 toxicities were reported for any of the adverse events. In addition, some adverse events, (e.g., constipation, cardiac general, and central nervous system) were not listed in the table due to Grade 1. All adverse events were controlled rapidly after appropriate corresponding treatments. No non-relapse mortality (NRM) occurred.

**Table 3:** Toxicities of grade 2-4 from ChiCGB.

Toxicities	Grade		
	2	3	4
Fever	7 (19.4%)	0	0
Vomiting	11 (30.6%)	4 (11.1%)	0
Mucositis	7 (19.4%)	6 (16.7%)	0
Rash	8 (22.2%)	3 (8.3%)	0
Diarrhea	12 (33.3%)	4 (11.1%)	0
Pneumonia	1 (2.7%)	0	0
Transaminase elevation	9 (25.0%)	7 (19.4%)	0

## Discussion

The results of this study suggest that ChiCGB represents a promising conditioning regimen for ASCT in patients with aggressive T-cell lymphoma. Compared with previously reported data, the outcomes in patients receiving ChiCGB were inspiring.

Although various conditioning regimens have been employed in ASCT for lymphomas, BEAM has been consistently favored as the preferred regimen (13). A large retrospective study confirmed that BEAM was the most effective regimen prior to ASCT (14). Clinical trials using BEAM or BEAM-like conditioning regimens in PTCLs have reported PFS rates of 30%–53% and OS rates of 39%–73% (15–18). In a retrospective study of GELTAMO/FIL, the PFS and OS rates were 63% and 74% in 103 patients with a median follow-up of 65.5 months (4). Other retrospective studies have reported varying outcomes with BEAM or BEAM-like regimens in PFS or event-free survival (EFS) ranging from 45% to 63% and OS ranging from 49% to 68% (**Table 4**) (20, 24–28). Notably, in an individual study, the survival rate of ASCT with BEAM as the conditioning regimen was found to be lower than that of conventional chemotherapy (24). In contrast, the new conditioning regimen of ChiCGB in this study yielded an encouraging PFS and OS of 81.2% and 92.0%, respectively, in all PTCL patients. For those in CR prior to ASCT, no relapses were reported at the last follow-up. We believe that the benefit of ChiCGB overcomes the negative impact of T-cell lymphoma on the prognosis compared with other types of lymphoma. For patients with T-LBL, previous literature has reported PFS rates of 46.9%–69% and OS rates of 58.3%–76% after undergoing ASCT (**Table 4**) (21–23). In this study, 11 T-LBL patients undergoing single ASCT after ChiCGB achieved both a PFS and an OS of 81.8% at three years, which was not inferior to the outcomes of tandem ASCT, although the sample size of this study was relatively small.

**Table 4:** Review of published studies of ASCT in PTCL and T-LBL.

Studies	Year	Conditioning	Cases	Study design	Disease types	Median Follow-up (month)	CR rate prior to ASCT (%)	PFS/EFS	OS	Ref.
Wu	2018	BEAM/BEAC/CBV/TBI+HD-CTX	79	Retrospective	PTCL	23.6	48.1	75.2% at 2 years	83.6% at 2 years	(19)
Garcia-Sancho	2022	BEAM	107	Retrospective	PTCL	66	100	63% at 5 years	74% at 5 years	(4)
Rodriguez	2003	BEAM	19	Prospective	PTCL	27	46	56% at 2 years	84% at 2 years	(20)
d’Amore	2012	BEAM/BEAC	115	Prospective	PTCL	60.5	NA	44%	NA	(16)
Mercadal	2008	BEAM/BEAC	17	Prospective	PTCL	38.4	NA	NA	<60% at 4 year	(17)
Wang	2020	BEAM/BEAC/CBV/TBI+HD-CTX	41	Retrospective	T-LBL	29	63.4	64.3% at 3 year	66% at 3 year	(21)
Song	2007	BEAM/TBI+CTX/TBI+CTX+VP-16	29	Retrospective	T-LBL	51	65.5	73% at 4 years	79% at 4 years	(22)
Liu	2021	BEAC+IAC	32	Prospective	T-LBL	37	52.8	60.4% at 3 years	66.3% at 3 years	(23)

BEAM, carmustine, etoposide, cytarabine, melphalan; BEAC, carmustine, etoposide, cytarabine, cyclophosphamide; CBV, cyclophosphamide, carmustine, etoposide; TBI, total body irradiation; HD-CTX, high-dose cyclophosphamide;VP-16, etoposide; IAC, idarubicin, cyclophosphamide, cytosine arabinoside; PTCL, peripheral T-cell lymphoma; T-LBL, T-cell lymphoblastic lymphoma; PFS, progression-free survival; EFS, event-free survival; OS, overall survival; NA, not available.

The synergistic effect of the chidamide, cladribine, gemcitabine, and busulfan combination was confirmed in previous research (9), which found that HDACi combined with CGB synergistically inhibited lymphoma cells in a certain order of medication administration. HDACi increased the sensitivity of genomic DNA to busulfan crosslinking *via* loosening and opening DNA. Cladribine and gemcitabine affected DNA synthesis and repair, leading to lymphoma cell apoptosis. All of these details serve as the theoretical and experimental basis for the effective conditioning regimen of ChiCGB.

Among the non-hematological toxicities, infection was the most frequently occurring non-hematological adverse event in patients receiving BEAM or BEAM-like regimens (29, 30). Other toxicities included mucositis, vomiting, and non-infective pulmonary complications (31, 32). n contrast, ChiCGB caused less severe toxicity, and all adverse events reported in this study were assessed as only Grade 3 or below. Compared with BEAM-like regimens, ChiCGB did not increase toxicity during the follow-up period. However, one patient with AITL developed EBV-positive DLBCL 28 months after ASCT, which is consistent with previous literature reporting that DLBCL secondary to AITL was often associated with EBV infection (33, 34). Furthermore, Zettl et al. discussed the onset pattern for these cases (35). The researchers thought that, in the setting of PTCL, B cells infected with EBV underwent atypical proliferation; together with the associated immune damage, that proliferation led to the development of B-cell lymphoma. On the other hand, one patient undergoing ASCT with another conditioning regimen also experienced DLBCL (36). The second tumor was therefore considered ChiCGB-independent. Overall, ChiCGB in our study was a safe and controlled conditioning regimen in T-cell lymphomas.

Nevertheless, our clinical trial had some limitations. First, as our study was a non-controlled trial where all included patients received ChiCGB as a conditioning regimen, no patients receiving other conditioning regimens were used as controls. The trial results were compared with other conditioning regimens in different periods. Second, although most patients participated in follow-ups for more than two years, the small sample size affected the statistical power, making us cautious in drawing conclusions. Third, a higher proportion of enrolled patients had IPI scores of 0, 1, 2, and 3, while IPI scores of 4 and 5 were absent, suggesting that patients with IPI scores of 3-5 were recruited to further strengthen the results.

## Conclusion

The results of the Phase 2 clinical trial suggested that ChiCGB was a potentially effective and well-tolerated conditioning regimen to improve the prognosis of patients with aggressive T-cell lymphoma. A randomized controlled clinical trial with a larger sample size on ChiCGB is needed for further investigation in the future.

## Data availability statement

The raw data supporting the conclusions of this article will be made available by the authors, without undue reservation.

## Ethics statement

The studies involving human participants were reviewed and approved by the ethics committee of West China Hospital. The patients/participants provided their written informed consent to participate in this study.

## Author contributions

JJ and ZL designed the study. PK, JL, XC, TD, TN, TL, ZL, and JJ recruited patients. QW, CZ, and CC took care of patients. QZ and HZ collected and analyzed the data, and wrote the paper. All authors contributed to the article and approved the submitted version.
